# Comparison of Repeatability and Stability of Residual Magnetic Field for Stress Characterization in Elastic and Plastic Ranges of Silicon Steels

**DOI:** 10.3390/s22083052

**Published:** 2022-04-15

**Authors:** Kun Zeng, Guiyun Tian, Bin Gao, Jia Liu, Yi Liu, Qianhang Liu

**Affiliations:** 1 School of Automation Engineering, University of Electronic Science and Technology of China, Chengdu 611731, China; bin_gao@uestc.edu.cn (B.G.); liujia617200@163.com (J.L.); liuyi1024@std.uestc.edu.cn (Y.L.); q.h.liu@std.uestc.edu.cn (Q.L.); 2 School of Engineering, Newcastle University, Newcastle upon Tyne NE1 7RU, UK

**Keywords:** repeatability and stability, elastic material status, plastic material status, magneto-optical Kerr effect microscope (MOKE), residual magnetic field (RMF)

## Abstract

Deep insights into microstructures and domain wall behaviors in the evaluation of different material statuses under elastic and plastic stress ranges have essential implications for magnetic sensing and nondestructive testing and evaluation (NDT&E). This paper investigates the repeatability and stability of residual magnetic field (RMF) signals using a magneto-optical Kerr effect microscope for the stress characterization of silicon steel sheets beyond their elastic limit. Real-time domain motion is used for RMF characterization, while both the repeatability under plastic ranges after the cyclic stress rounds and stability during relaxation time are studied in detail. The distinction between elastic and plastic materials is discussed in terms of their spatio-temporal properties for further residual stress measurement since both ranges are mixed. During the relaxation time, the RMF of the plastic material shows a two-stage change with apparent recovery, which is contrasted with the one-stage change in the elastic material. Results show that the grain boundary affects the temporal recovery of the RMF. These findings concerning the spatio-temporal properties of different RMFs in plastic and elastic materials can be applied to the design and development of magnetic NDT&E for (residual) stress measurement and material status estimation.

## 1. Introduction

Since the metal magnetic memory (MMM) method was proposed by Dubov [1], it has attracted researchers’ attention as a passive detection approach. This technique shows a promising prospect in early defect detection and residual stress assessment, and the advantages are described in [2,3]. However, the problem with measuring the characteristic stress with the MMM is that it mainly focuses on the basic experimental research without considering the material status or microstructure influence. Meanwhile, the stable fluctuation stage of the magnetic signal in the failure process cannot effectively characterize the fatigue damage state. In particular, when the ferromagnetic material is under stress that exceeds the yield strength, it leads to a region where elastic and plastic deformation coexist, and this affects the material properties. However, it can be difficult to identify the material status and macroscopic magnetic parameters of the materials in the elastic and plastic ranges, which can cause the inaccurate assessment of stress. Therefore, the design of new sensing structures and the extraction of new characteristic parameters are important for the determination of the material status and the evaluation of the residual stresses in the nonlinear region. Roskosz et al. [4] combined a RMF with Barkhausen noise characteristics and impedance components in an in-series LCR circuit to evaluate the degree of plastic deformation of X2CrNi18–9 steel and the extracted diagnostic signal features, which can distinguish between the two ranges of strain variability. Liu et al. [5] calculated and analyzed the variation trend of the triaxial weak magnetic signal with stress and propagation distance in the pipeline, which showed that there is a linear relationship between weak magnetic signals and stress, and the weak magnetic signal reverses with the increase in stress. At the same time, the weak magnetic signal attenuates exponentially with the increase in transmission times. Leng et al. [6] showed that the change in MMM signal is easily affected by the transition stage of elastic–plastic deformation. This result provided a feasible and straightforward new method for detecting macroscopic yield and early plastic deformation in ferromagnetic materials. Su et al. [7] established a strain-based Jiles–Atherton hysteretic model under low cyclic loading. This model provided a direct way to quantitatively evaluate low cycle fatigue cumulative plastic damage in low-carbon steel. Meijers et al. [8] pointed out that the irreversible change in structural magnetization caused by plastic deformation is different from that caused by elastic deformation. For the MMM signal under stress loading and after unloading, Wang et al. [9] used the combination of online loading and unloading measurements to obtain the characteristics of the signal curve. The results showed that the MMM signals in the elastic and plastic stages have different trends and provide a reference for evaluating and monitoring the stress state of ferromagnetic materials. In conclusion, MMM has advantages in stress assessment, but its stability and repeatability still require attention. Although the repeatability and stability problems were studied in [10], this study was limited to elastic materials. In order to draw comparisons with plastic materials, there needs to be an in-depth study on the repeatability and stability of RMFs for residual stress characterization as the elastic and plastic material statuses could be mixed.

In addition to passive methods, active nondestructive testing (NDT) techniques have illustrated different characteristics of elastic and plastic materials. Liu et al. [11,12] studied the relationship between the microstructure of materials and the transient eigenvalues of magnetic Barkhausen noise (MBN) under elastic and plastic stresses. The results showed that the eigenvalues of MBN have different trends in the elastic and plastic ranges. Xie et al. [13] studied the relationship between the electromagnetic properties and plastic deformation of 304 austenitic stainless steel. The results showed that the increase in plastic deformation leads to a decrease in electrical conductivity and the improvement of the magnetic properties of materials. The effects of fatigue damage on electromagnetic properties and microstructure analysis were also confirmed in their research. Klyushnikov et al. [14] studied the temperature dependence of the acoustic and electromagnetic properties of AISI321 austenitic steel under plastic deformation, and the results could distinguish damage from martensitic transformations. Matsumoto et al. [15] used the eddy current magnetic characteristic method (EC-MS) to study the trajectory of the eddy current signal before and after the yield point, which showed that the residual strain is caused by plastic deformation, which has a noticeable influence on the EC-MS. Pei et al. [16] proposed and developed a new noncontact and uncoupled plastic strain measurement method based on EMAT, and the degree of polarization of the Rayleigh wave was measured and applied to the measurement of plastic strain. The results showed an excellent linear relationship between the relative change in the Rayleigh wave and plastic strain.

It can be seen that different NDT methods distinguish the characteristics of elastic and plastic deformation, while we still lack a sufficient explanation from the perspective of microstructures. In addition, there exist deficiencies in the identification of material status and stress status. However, active and passive detections require us to improve the existing sensing modes by combining macroscopic parameters and microstructural features to differentiate between elastic and plastic materials effectively. In particular, the acquisition of features with better repeatability and stability is the main focus. In this work, the RMF signals for stress characterization in the elastic and plastic material statuses of silicon steel sheets is studied. Different RMF behaviors after removing magnetization under different stresses of different material statuses are evaluated and applied to solve the problem of the influence of MMM on quantitative analyses of stress characterization, particularly as residual stress has mixed plastic and elastic ranges within the material.

This paper is organized as follows: after the introduction, the method and experimental equipment are described. Then, the magnetic domain patterns in different material states, the repeatability of a RMF under cyclic stress rounds, and the stability during the relaxation time after the stress was released are studied and discussed. Finally, our conclusions are summarized and future work is discussed.

## 2. Method and Experimental Setup

**Figure 1:**
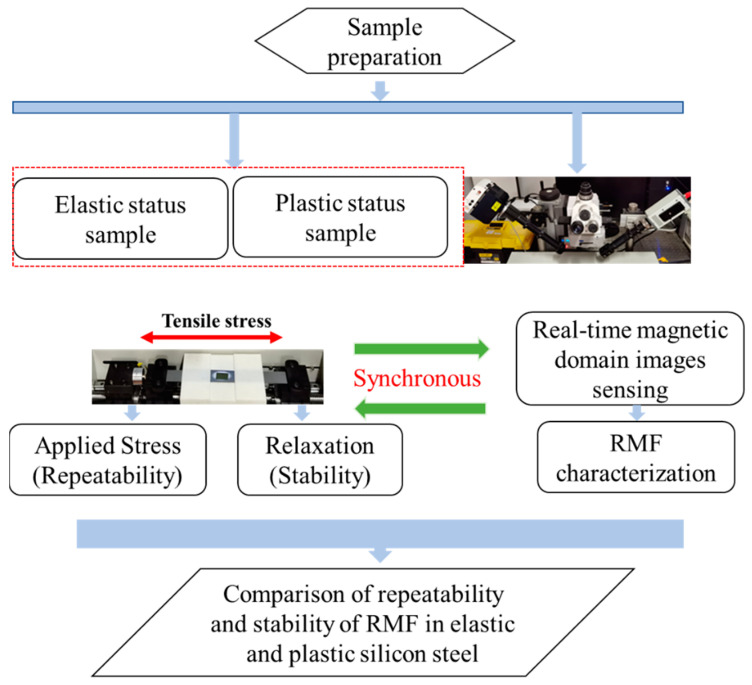
Schematic diagram of the experiment.

The changes in magnetization after stress is applied can either be reversible or irreversible, depending on the domain motion process. Generally, reversible and irreversible processes are carried out simultaneously, so magnetization cannot be restored to its initial state after stress is applied. After the applied stress exceeds the yield strength, the material properties will change significantly and the irreversible domain motion will dominate. A schematic diagram of this study is shown in Figure 1. This study involved preparing plastic range materials and conducting a repeatability experimental study in line with previous work [10]. After sample preparation, the magnetic domain motion of the samples in different material statuses were recorded by in situ MOKE in real time under different stress statuses. A relationship model between the domain motion and the RMF was established, and the real time variation in domain motion under cyclic stress rounds and during the relaxation time was analyzed by the characterized RMF. In addition, the effects of grain boundary and historical loading on domain motion were studied. The study’s method and experimental setup are shown in Figure 1. The figure illustrates different elastic and plastic samples of silicon steel measured by RMF distribution using a magneto-optical Kerr effect microscope (MOKE) with microstructure information.

### 2.1. RMF Characterization Method and Domain Motion Behaviors

Under the excitation of the geomagnetic field, a surface leakage magnetic field was generated in ferromagnetic materials due to the inhomogeneity of the materials, thus forming MMM signals. The RMF is the normal component of the MMM signal and can be mapped on magneto-optical indicator film (MOIF) placed on the surface of the specimen. As shown in [10], two features are proposed to characterize the RMF based on domain images. The mean value (MV) characterizes the magnitude of the RMF, and as the domain wall is much smaller than the domain width, the domain rotation plays a decisive role in the MV. The distribution of RMF is characterized by the angular second moment (ASM) of the MOKE signal intensity, which is determined by domain wall displacement. The magnitude and distribution of RMF under stress can be characterized in real time through those two features.

As a positive magnetostriction material, silicon steel is dominated by the 180° domain without an external field. Meanwhile, there are a few 90° domains to ensure the minimum demagnetization energy. When tensile stress parallel to the rolling direction is applied to the specimen, the dynamic equilibrium of the magnetic domain is broken, and the magnetoelastic energy *E* can be expressed as

(1) $$E=-\frac{3}{2}\lambda \sigma cos^{2}\theta$$

where λ is the saturation magnetostriction coefficient, σ is the applied stress, and θ is the angle between the stress and magnetization directions. Under tensile stress, the 180° domain increases, while the area of the 90° domain decreases until it disappears, and the system’s energy reaches the minimum when θ is 0° or 180°. Under compressive stress, the magnetic domain area of 180° decreases and the magnetic domain of 90° (270°) increases. When the compressive stress is great enough, all of them become 90° domains, and the system’s energy is at its lowest.

Magnetic domains in different regions have different motion characteristics under stress as a result of the inhomogeneity and the hindrance of pinning, and the motion characteristics at the grain interior are different from those at the grain boundary. Cyclic stress rounds will also break through the hindrance of structural defects, such as grain boundaries, Goss texture, and dislocation density, and cause the accumulation of residual stress. Thus, after the stress is removed, the stress-induced magnetic domain motion is ‘memorized’. Most of the magnetic domain motion caused by elastic stress can be recovered. However, the magnetic domain movement caused by plastic strain produces residual compressive stress in the deformation region. To minimize the system’s total energy, the orientation of the magnetic domain is transformed and new magnetic domain patterns are generated.

### 2.2. Experimental Setup

**Figure 2:**
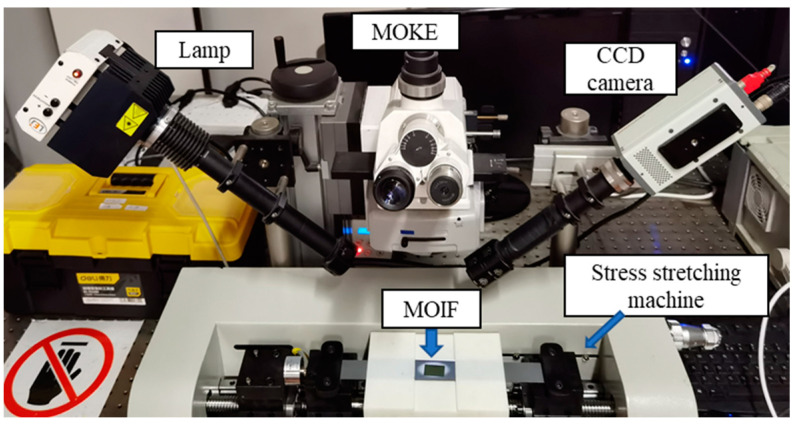
Magnetic domain acquisition and mechanical system.

The experimental setup is shown in Figure 2. This system contained a stress stretching machine based on PLC control, an in situ MOKE, and a magneto-optical indicator film (MOIF). In this MOKE system, with the help of MOIF, magnetic domains were observed without any surface treatment of the samples, and all the domain dynamic behaviors were recorded in real time by a digital CCD camera with a sampling rate of 16 Hz. Referring to the national standard of the People’s Republic of China, GB T228.1-2010, the experimental temperature was kept at 20 degrees centigrade, and the relative humidity was maintained at 50%. The above two environmental parameters were kept constant to reduce external influence.

A specially designed stress stretching machine can maintain a stable loading/unloading rate of 0.5 mm/min while ensuring that the domain images captured in MOKE are in the same position. The loading process was under a stress-controlled mode in the elastic range, and after exceeding the yield strength, the displacement control mode was adopted. The unloading process was stress-controlled in both ranges. This machine can automatically carry out cyclic stress rounds and maintain the set stress value between each round for some time. The maximum tensile stress value of the equipment is 3430 N (for the samples in this study, the tension reached about 500 MPa), which met the stress requirements of this study.

### 2.3. Sample Preparation

High grain-oriented (HGO) silicon steels of grade 23Z110 produced by Nippon Steel Corporation were used as samples in this paper. Due to its large grain size, clearly visible grain boundary, and easy-to-see magnetic domain structure, the magnetic domain motion and the influence of grain boundary can be studied easily. The material’s length is 300 mm, the width is 30 mm, and the thickness is 0.23 mm. The chemical components are shown in Table 1. The yield strength is 320 MPa. Before the experimental procedures, all samples were demagnetized without any external stress.

The maximum stress of the sample (named sample 1) in the previous study [10] was 87 MPa, after which this sample continued to maintain an elastic material status. To compare the repeatability and stability in different material statuses, three other samples (sample 2, sample 3, and sample 4) are used in this paper. The domain widths of those samples are similar, but the location and density of grain boundaries are different. The sample was first loaded to a preset tensile stress value (PSS) and maintained for 5 s, and then the applied stress was removed so that the material returned to the no applied stress status. At this time, the magnetic domain images were selected for the RMF signal characterization. After holding for 5 s, the above procedure was repeated with another PSS. The PSS was increased at 45 MPa stress intervals. When the yield strength was exceeded, the three samples continued to be stretched to 1.5% strain. After holding for 5 s, the stress was removed so that the material returned to the no applied stress status. At this time, one stress round was formed and the samples were in the plastic material status.

In this paper, several stresses were applied at different time intervals. The time intervals between two adjacent stress rounds were 20 s, 3 min, 20 min, 8 h, and 24 h. By analyzing the changes in the RMF during cyclic stress rounds, the repeatability of the RMF in plastic materials was identified. Following this, the RMF changes during the eight-hour relaxation time after the removal of different stresses were tracked continuously and the stability characteristics were extracted. Furthermore, in order to compare the characteristics of the plastic material when it had just become plastic and after it had been plastic for a long time, after the cyclic stress rounds, the specimen was placed in a sealed box for 45 days. After that, 1.5% stress was applied to the sample again, and the change during relaxation time was tracked.

## 3. Results and Discussion

Through the comparison of the RMF in elastic and plastic status samples under different stress statuses, it was found that there are significant differences between them in the following three respects.

### 3.1. Magnetic Domain in Different Material Statuses

**Figure 3:**
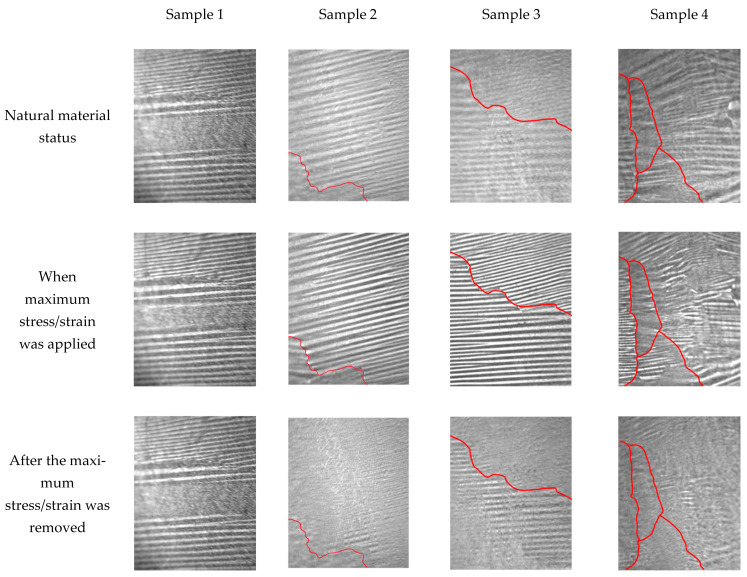
Domain images of samples in different material statuses. The red lines represent the grain boundaries.

Figure 3 shows the domain images of samples in different material statuses under different stress statuses, and the red lines represent the locations of grain boundaries. The maximum stress of sample 1 was 87 MPa, and the maximum strain of samples 2, 3, and 4 was 1.5%. In the three stress statuses, the magnetic domains of all samples exhibited visible changes, especially when the maximum stress/strain was applied; the magnetic domain images were clearer, and the magnetic domain textures were denser. Stress-induced magnetic domain motion is the cause of this phenomenon. In the loading process, the original supplementary domain rotated towards the orientation of the striped domain, and the domain wall of the striped domain was tightened at the same time. Because of the small stress, the change in the magnetic domain of sample 1 was not as apparent as that of the other samples. After the stress was removed, sample 1 was in the elastic material status, in which most of the magnetic domains returned to the initial state and a small portion of them did not recover. In contrast, the other three samples were in plastic material status. More magnetic domains did not recover to the initial status in those samples and even produced domain transitions due to the residual compressive stress in the elongated part. At this time, those residual compressive stresses lead to the transition of the magnetic domain from the striped domain to the transverse domain, resulting in a change in the domain orientation. Therefore, the magnetic domain in different material statuses is significantly different.

After the 1.5% strain, samples 2, 3, and 4 were all in a plastic material status, but the changes in the magnetic domains were different. Sample 2 had a large grain, and the grain boundary was located at the lower left of the grain. Most of the magnetic domains in the grain interior were transformed in this sample, and only the part near the grain boundary still maintained the original striped domain structure. There were two grains in sample 3, and the grain boundary was located in the middle of the sample. The microstructure of sample 4 was the most complex, containing four grains of different sizes. Similar to sample 2, the magnetic domain at the grain interior is easy to change when the samples are in the plastic material status. The existence of the grain boundary hinders the domain motion, which makes the striped domain easier to retain at the grain boundary. Those domain images can qualitatively observe these changes in magnetic domains, and the quantitative characteristics can be calculated by characterizing them with RMF signals. Thus, the repeatability and stability of the domain motion of plastic materials under different stress statuses were analyzed in order to find relevant features for stress characterization—for materials in the plastic range in particular [17,18].

### 3.2. The Repeatability of RMF under Cyclic Stress Rounds in Different Material Statuses

**Figure 4:**
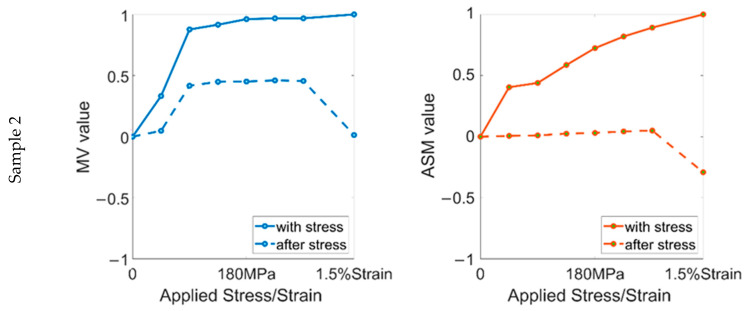

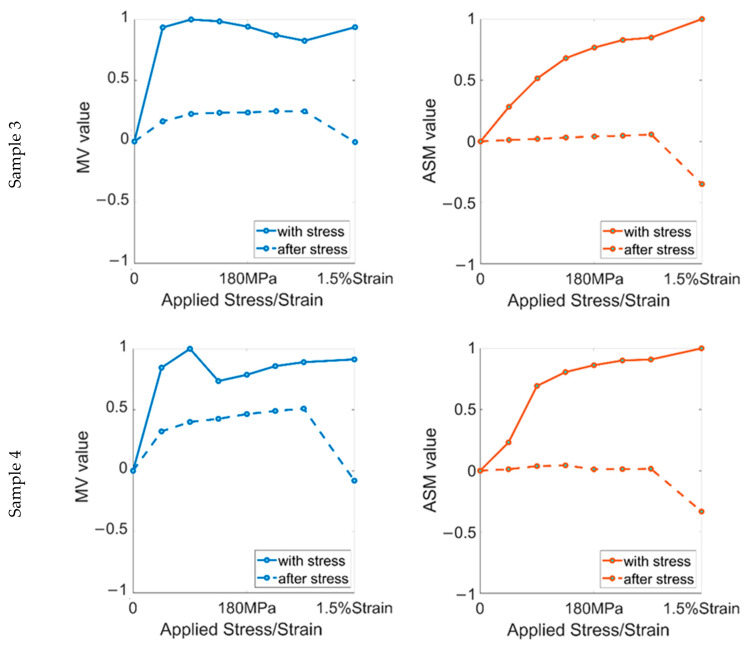
The relationship between RMF signal and applied stress/strain in the first stress round.

The relationship between the RMF and the applied stress/strain in the first stress round is shown in Figure 4. The results clearly show that in the elastic range, both components of the RMF signal increased with stress. In sample 1 and the low-stress ranges (0–90 MPa) of the other samples, the MV parameters increased linearly with stress. However, when the stress increased above 90 MPa, the sensitivity of the MV parameters decreased, even to the point where it hardly changed with increasing stress. In contrast, the ASM parameters of all samples were linearly related to the stress throughout the elastic range. This can be traced back to the stress-induced magnetic domain motion mechanism. In the low-stress range, the stress leads to the domain rotation and the domain wall displacement simultaneously. However, when a threshold stress value is reached, the supplementary domain is rotated entirely to the direction of 180°, and the magnetic domain rotation is limited. At this time, the magnetic domain wall displacement becomes the dominant motion.

After stress lower than 90 MPa was removed, similar to the change in sample 1, the behavior of the MV parameter was still proportional to stress. However, after a larger elastic stress was removed, the linear change in the MV was no longer seen and the MV remained at an almost constant value. In contrast, the linearity of the ASM with stress was maintained throughout the elastic range. Nevertheless, the stress sensitivity was significantly reduced after the stress was removed, and its change with stress was very weak. The results show that, after the applied stress reaches a threshold, a considerable part of the supplementary domain cannot rotate to the initial state after the elastic stress. With elastic stress, the domain wall displacement is carried out all the time, while most of them return to the initial state after the stress is removed.

After the yield strength was exceeded, the MV and ASM parameters continued to follow the trend of the elastic range. However, after the 1.5% strain was removed, the magnetic domains of the samples underwent a significant transformation. This also led to a rapid decrease in the two components of the RMF signal, which are the opposite of the elastic range. After 1.5% strain, the ASM reached a negative value for all samples and decreased by about 30–35% compared with the initial value. However, the change in the MV parameters was different due to the presence of different microstructures. The MV parameter of sample 2 was slightly larger than the initial value, while sample 3 was roughly the same as the initial status. For sample 4, the MV was smaller than the initial value. It can be seen that after becoming a plastic material, the effect of the microstructure on the domain wall displacement is similar, resulting in a similar change in the RMF’s distribution. From the results in the elastic range, we can see that the MV parameter is more sensitive to stress, which shows that the residual stress in sample 4 is different from that of the other samples. Due to the accumulation of dislocations at the grain boundary caused by plastic deformation, various nonuniform deformations occur when the dislocations pass through the grain boundary and slip between multiple grains. These become the main reason why the samples with high grain boundary density have more microscopic residual stresses (type II and type III residual stresses).

**Figure 5:**
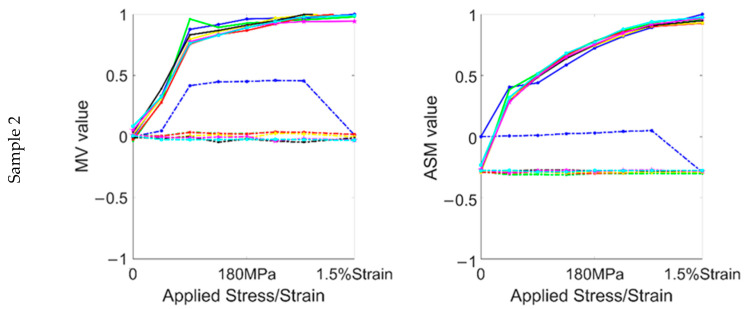

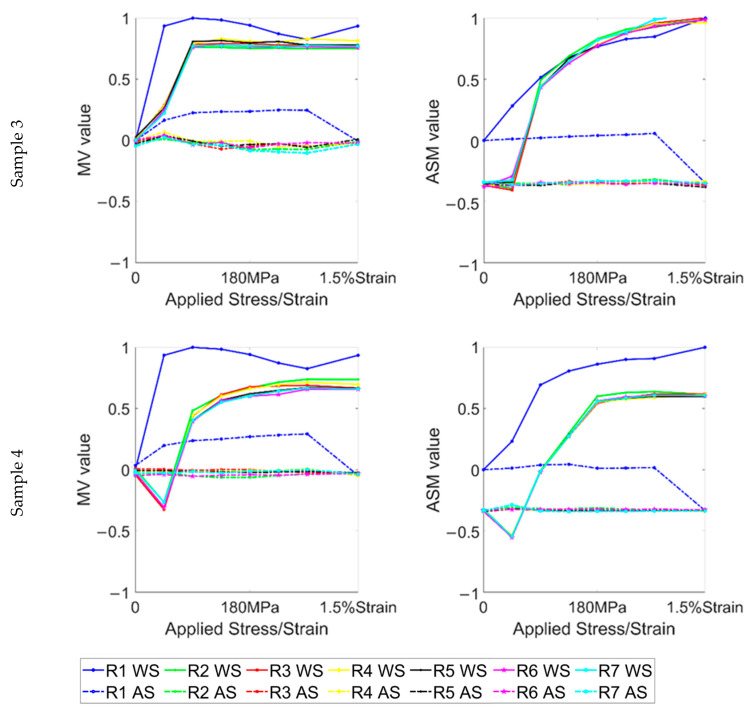
The relationship between RMF signal and applied stress/strain in the cyclic measurements.

After the maximum stress in the first round, sample 1 was still in the elastic material status. In the ensuing cyclic stress rounds, the MV and ASM parameters of this sample maintained similar values during and after the same stress. When the maximum load in the first round was plastic strain, there were some differences compared with sample 1. After the maximum strain, the samples became a plastic material, which destroyed the initial magnetic domain state of the sample. The values of the MV and ASM parameters in different samples regarding the repeatability in different cyclic stress rounds are shown in Figure 5. The cases with stress (WS) and after stress (AS) are shown. In the cyclic stress rounds with different time intervals, the changes in the two components of the RMF were small, both with and after the same stress. This result, together with the results of sample 1, shows that after the sample reaches the plastic material state, stress less than the historical maximum loading cannot change the current material status.

Furthermore, after reaching the plastic status, it can be seen that there is not much difference in the MV and ASM parameters after the same stress in different rounds. It should be noted that in sample 3, starting from the second cyclic stress round, when the sample was under 45 MPa of stress, the change in the RMF was unique compared with the other stresses, and the phenomenon in sample 4 was more distinct. This is related to the residual compressive stress after plastic deformation. After the applied stress of the plastic material is removed, the resultant force of the plastic material is the direction of the compressive stress. In the loading process of tensile stress, the resultant force direction of the material changes from the compressive stress direction to the tensile stress direction. Because the resultant force is less than the applied tensile stress, the RMF of the sample with the same stress cannot reach the value before it becomes a plastic material. A larger grain density is accompanied by more residual compressive stress, and greater tensile stress is needed to change the direction of the resultant force. Therefore, this phenomenon occurs in sample 4 with a higher grain density.

Through the statistical analysis of these data, the standard deviation (STD) of the RMF value in different measurements (except the first measurement) after the same stress was calculated and is shown in Table 2. The STD of the MV parameters of the three plastic samples is always larger than that of the ASM, indicating that the domain wall displacement is more stable than the magnetic domain rotation after different stress statuses. However, for sample 1 in elastic material status, the minimum STD value of the MV in different regions is 0.0426, and the minimum STD value of the ASM is 0.0307. In contrast, the STD of the RMF of plastic materials is smaller. This shows that after reaching the plastic material status, the repeatability of the magnetic domain motion is better than that of elastic materials. In this case, due to the effect of residual stress, the disordered domain motion is limited, which leads to the better repeatability behavior of the plastic materials after cyclic stress rounds.

### 3.3. Stability of RMF during Relaxation Time after Stress Was Removed in Different Material Statuses

In sample 1 [10], the magnetic domain does not reach a constant value instantly and, the variation in the RMF after the stress is removed can be divided into two parts. The first part, which is defined as B time in the reference, starts from the moment the stress is removed and lasts until a few seconds later, and rapid recovery of the domain mainly occurs in this part. The second part is called C time, and continuous magnetic domain motion is carried out in this part, but the motion appears to be oscillatory and stable. Generally speaking, this shows a changing trend of ‘recovery–stability’. During the relaxation time of sample 1, the change amplitude of the MV is more than 10%, and the change amplitude of the ASM is about 5%. However, after the 1.5% strain is removed, the MV and ASM parameters of the plastic samples change in a manner more complex than those of sample 1.

**Figure 6:**
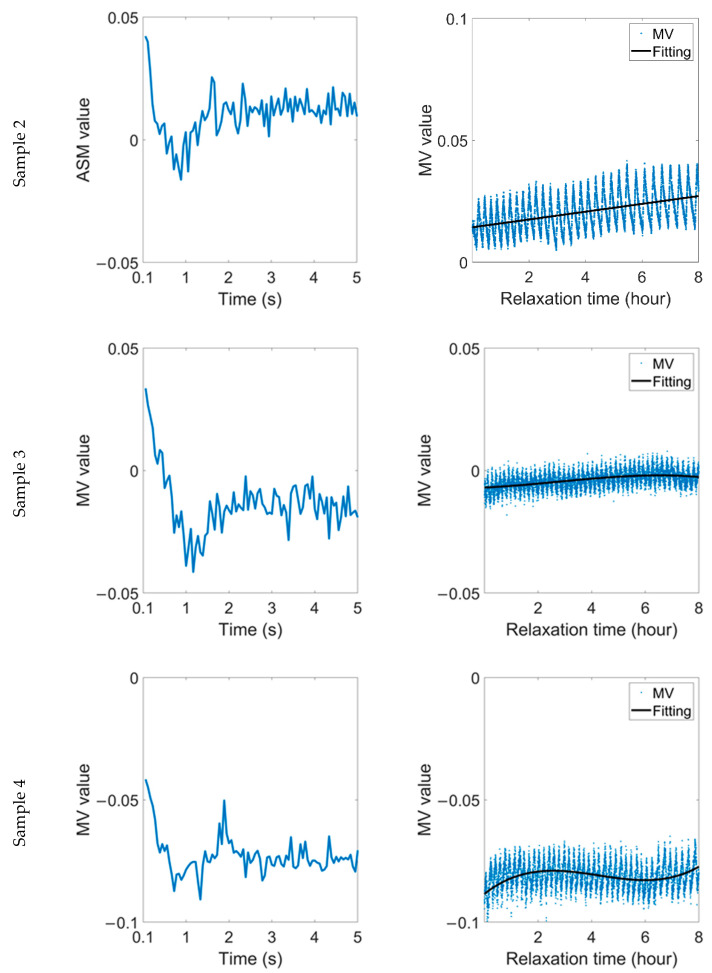
Variation in MV parameter during relaxation time after 1.5% strain was removed.

After the end of the cyclic stress rounds, the changes in magnetic domain were continuously tracked for eight hours after the stress was removed. Figure 6 shows how the MV parameter changed after the 1.5% strain was removed. The first column shows the change in B time after the stress was removed; the second column shows the change in the MV during the 8-hour relaxation time (C time). The MV parameter of these samples presents a rapid decline after the strain is removed, and then increases again after about 1 s. Finally, the MV parameter reaches a relatively stable state within 2–3 s. During the relaxation time, through the fitting curve, it can be seen that the MV mainly increases, and this phenomenon is similar to the change that occurs within 5 s after the applied stress is removed.

**Figure 7:**
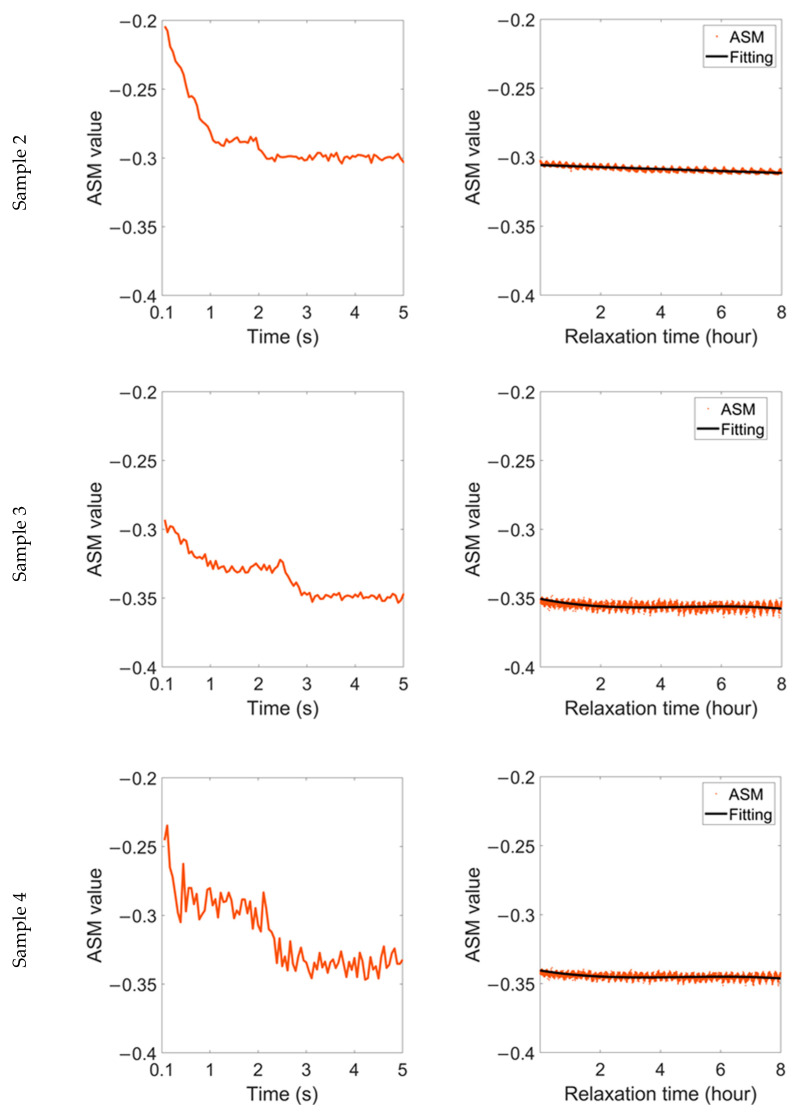
Variation in ASM parameter during relaxation time after 1.5% strain was removed.

The variation in the ASM parameter during relaxation time after 1.5% strain is shown in Figure 7. Similarly, the ASM parameter also shows a ‘two-stage’ change. It reaches a steady value about 1 s after the strain is removed, but the transient stability is broken after a few seconds. After that, the ASM continues to decline and finally reaches an oscillatory stable value again.

Even under the same stress and material status, samples with different microstructures behave differently during the relaxation time. The RMF oscillation of sample 2 is lighter than that of the other two samples, accompanied by a lower frequency, and the fitting curves of the MV and ASM in this sample are linear during relaxation time. In comparison, for the samples with high grain boundary density, the accumulated residual stress is released slowly during the relaxation time, resulting in more significant magnetic domain motion. As a result, the linearity of the fitting curve in sample 3 is lower, while the fitting curve in sample 4 shows a nearly sinusoidal distribution.

Although the change range of the RMF of different samples is different, it can be maintained at ±2% or less. In comparison, during the relaxation time of elastic materials, the change range of the ASM is similar. However, the change range of the MV can be up to ±10%, and the RMF of plastic materials is more stable during the relaxation time.

**Figure 8:**
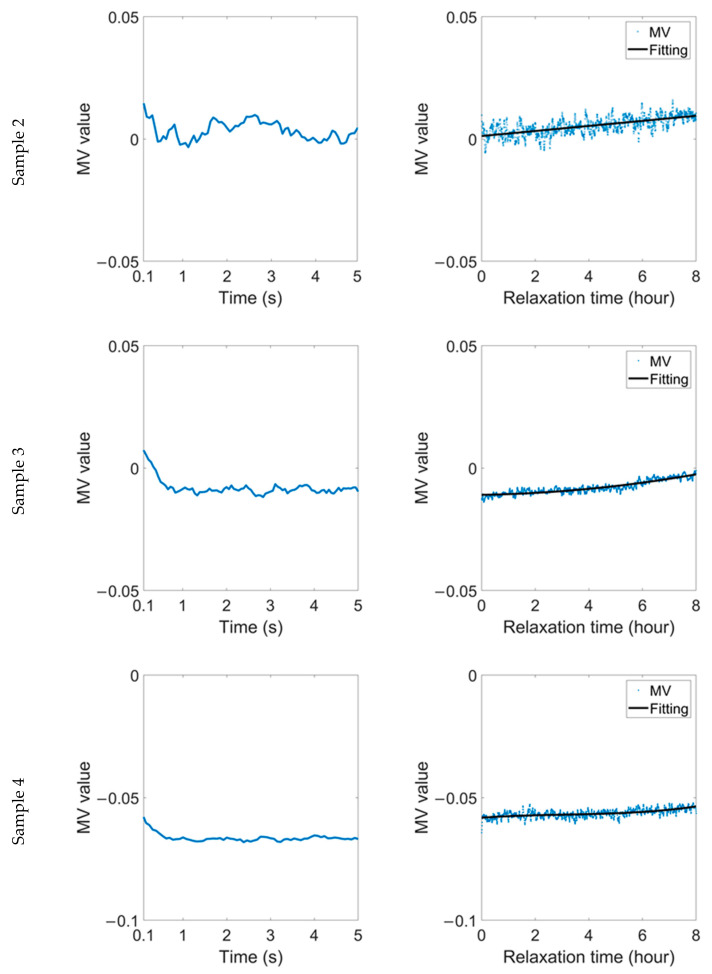
Variation in MV parameter during relaxation time after 1.5% strain was removed after the samples had been in plastic status for 45 days.

After placing the sample, which had been in plastic status, in a sealed plastic box for 45 days, the stress was applied to the sample again. After the stress was removed, the change in the RMF was tracked. From the variation in the MV parameter in Figure 8, the original trend of rapid change in the MV during B time slows down significantly. In C time, the MV still shows an inevitable recovery trend, but, taking the fitting curve as a reference, the oscillation amplitude becomes very subtle. In particular, in sample 4, the original sinusoidal change, is converted to an approximately linear change.

**Figure 9:**
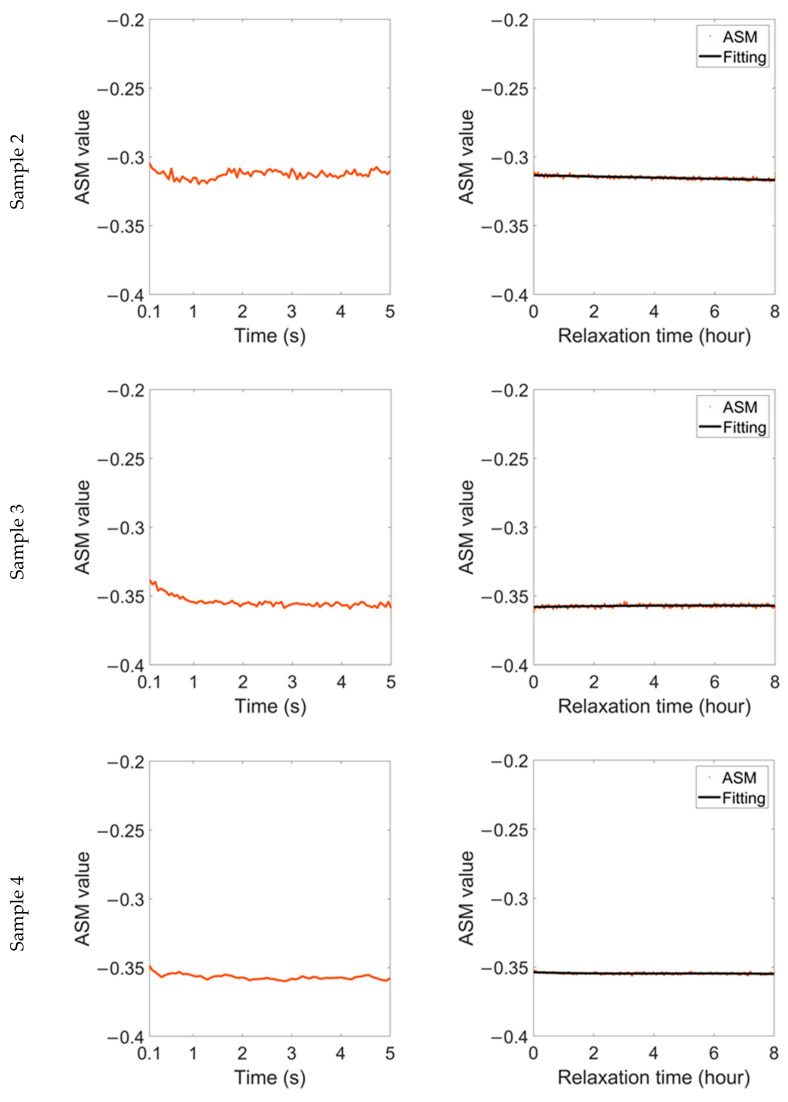
Variation in ASM parameter during relaxation time after 1.5% strain was removed after the samples had been in plastic status for 45 days.

The variation in the ASM shown in Figure 9 also highlights the improvement in the stability of the sample after 45 days in a sealed box. In B time and C time, the ASM of each sample shows a similar trend to the MV. The variation in the two parameters shows that the stability of plastic materials will be enhanced after being placed for a long time, and it is less sensitive to stress. There will be a process of stress relief inside the material during this time, although it is not apparent at room temperature. The residual stress caused by plastic deformation will always act on the material, resulting in the magnetic domain always being in the transverse pattern. Compared with an elastic material and new plastic materials, this kind of ‘old’ plastic material has better stability and lower stress sensitivity.

## 4. Conclusions and Future Work

In this paper, a comprehensive comparison of the residual magnetic field is put forward for domain stress characterization in the elastic and plastic statuses of silicon steels based on a previous study [10]. As preparation, a stress stretching machine and in situ magnetic domain observation system were set up to obtain real-time images of magnetic domains under different stress statuses. This paper explores the magnetic domain patterns, the repeatability after cyclic stress rounds, and the stability during relaxation time after stress was removed in different material statuses. Our results led us to the following conclusions:

(1) When stress/strain is applied, the domain image becomes clearer and the domain width becomes narrower. After the stress in the elastic range is removed, the magnetic domain returns towards the initial state, which mainly consists of striped domains. However, after the material reaches the plastic status, the striped domains change into new transverse domains. As a result, there is a significant difference between the magnetic domains in the elastic and plastic ranges.
(2) When a small stress (0–90 MPa) is applied, this induces the domain rotation and domain wall displacement to proceed synchronously. When the applied stress continues to increase, the supplementary domain is completely rotated, and only the domain wall displacement continues. Thus, the RMF linearly increases with stress but the MV has almost no stress sensitivity to stress exceeding 90 MPa. After the stress is removed, the magnetic domain rotation and the domain wall displacement cannot return to the initial state, resulting in a change in the RMF value. The elastic stress increases the RMF, while the RMF changes in the opposite direction after the plastic strain is removed. In the cyclic stress rounds, because the small stress cannot change the current material status, there is no substantial change in the RMF under different stresses. Compared to the STD of the RMF after stress in each elastic stress round, materials in plastic status have better repeatability after cyclic stress rounds.
(3) For samples that have just reached a plastic status, the MV decreases at first then increases to achieve oscillatory stability. The ASM has transient stability for a while in the reduction process and then falls to the state of oscillatory stability again. In the eight-hour relaxation time after the stress is removed, on the whole, the MV shows an upward trend, while the ASM shows a downward trend. For samples that have been a plastic material for a long time, the variation in the RMF after stress is more stable and less sensitive to stress. However, due to the limitation of residual stress caused by plastic deformation, the oscillation amplitude of the RMF of plastic materials is much smaller than that of elastic materials, and the stability of the RMF of plastic materials is more prominent.
(4) Due to the accumulation of microscopic stress at the grain boundary, the materials in a plastic status with high grain boundary density produce more residual compressive stress and limit the domain motion behaviors, resulting in a smaller change range of the RMF with the highest grain boundary density. Therefore, plastic materials with high grain density have better stability and repeatability.

The results of this paper show that the material status has different levels of repeatability in cyclic stress rounds and stability during the relaxation time after stress was removed. These results provide the RMF with a feasible method of (residual) stress characterization, e.g., pipelines after MFL, where material status may be a mix of elastic and plastic, and the conclusions derived from the results are very useful for future design when exploring the nonlinear region. In future work, these findings can be applied to identify the material state during stress assessment and provide theoretical guidance for the design of sensing structures for stress detection using the MBN/MMM method.

## Figures and Tables

**Table 1:** Chemical components of the silicon steels expressed as weight percentages.

Fe	Si	C	Mn	P	S	Al
Balance	3.00–5.00	0.06	0.15	0.03	0.25	5.10–8.50

**Table 2:** Comparative results of the repeatability of RMF in different measurements.

		0	45 MPa	90 MPa	135 MPa	180 MPa	225 MPa	270 MPa	1.5% Strain
Sample 2	MV	0.007	0.012	0.024	0.029	0.020	0.037	0.035	0.021
	ASM	0.007	0.014	0.012	0.015	0.011	0.012	0.010	0.010
Sample 3	MV	0.019	0.019	0.013	0.024	0.029	0.027	0.028	0.014
	ASM	0.013	0.007	0.009	0.011	0.010	0.012	0.013	0.015
Sample 4	MV	0.024	0.015	0.021	0.023	0.024	0.018	0.019	0.010
	ASM	0.005	0.015	0.007	0.008	0.009	0.006	0.005	0.005
